# Supplementary material for: Dynamic regulation of GDP binding to G proteins revealed by magnetic field-dependent NMR relaxation analyses
Source: Nat Commun. 2017 Feb 22;8:14523. doi: 10.1038/ncomms14523 (PMC5322562; doi:10.1038/ncomms14523)
Supplement: Supplementary Information — Supplementary figures and supplementary references. [file ncomms14523-s1.pdf]

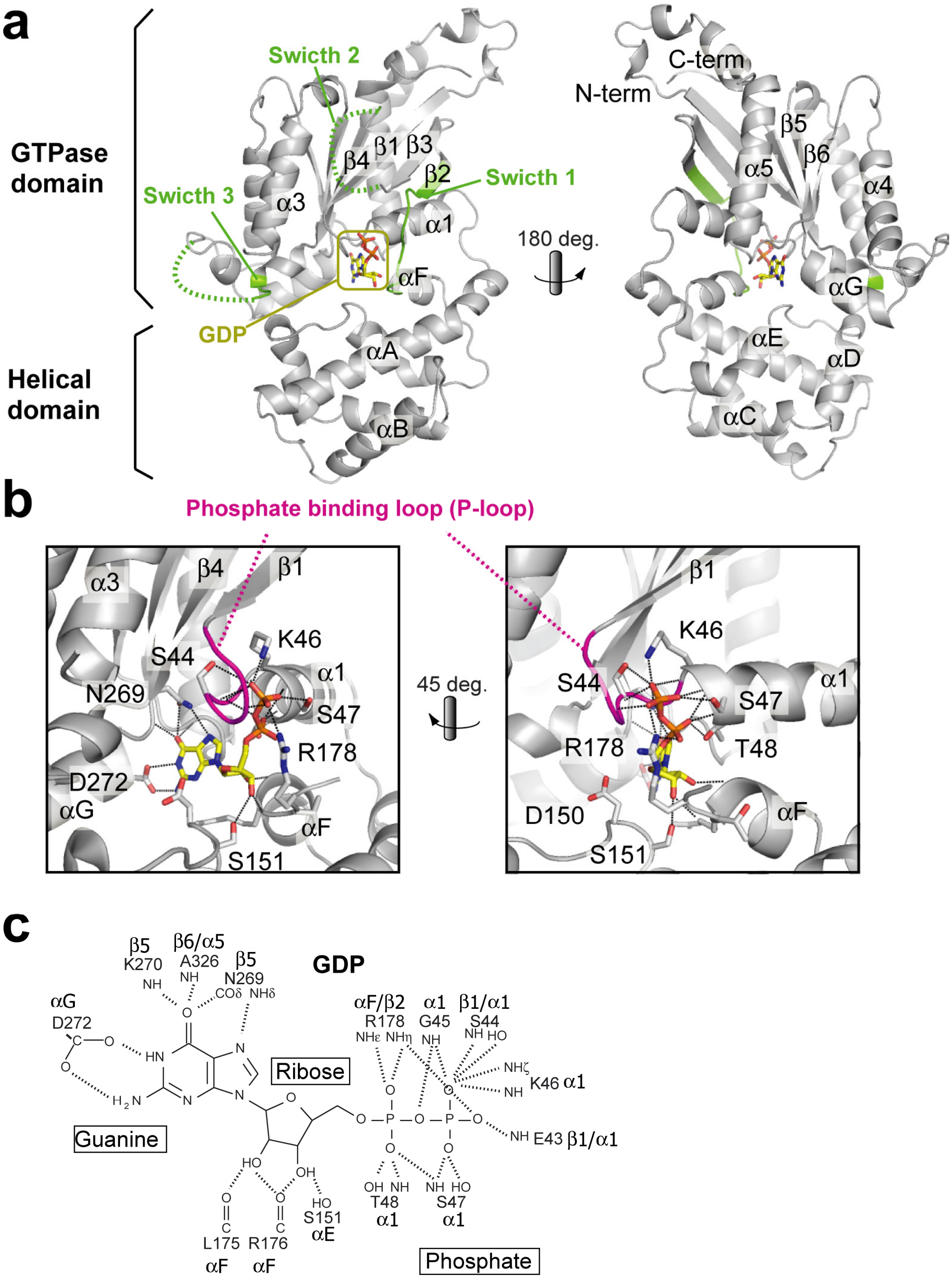

Supplementary Figure 1 Structure of Gα·GDP

(a) Crystal structure of Gα·GDP (PDB ID : 1GDD)[1]. The switch regions are colored green.

The regions lacking the electron density are depicted by dashed lines. (b) An enlarged view of the GDP-binding region. The phosphate binding loop (P-loop) is colored magenta. (c) A schematic representation of the Gα-GDP interactions.

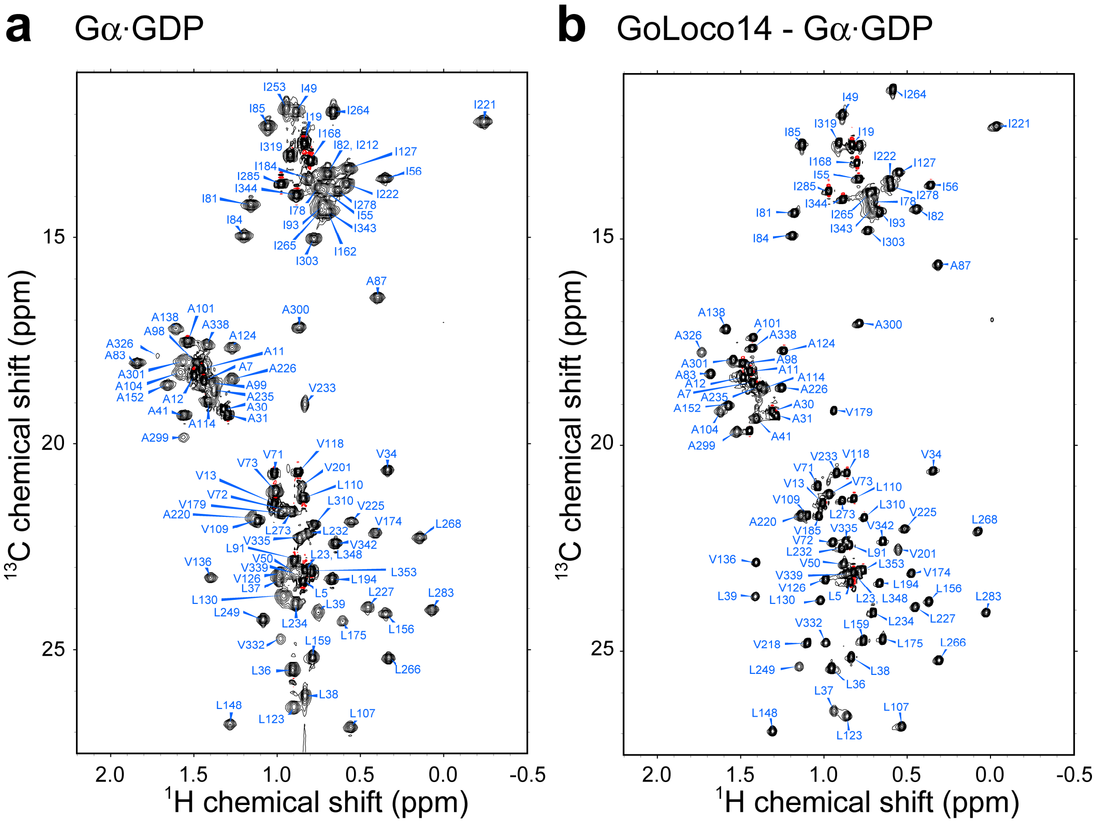

Supplementary Figure 2 ^{1}H-^{13}C HMQC spectra of Gα·GDP

(a) ^{1}H-^{13}C HMQC spectrum of 200 μM {u-^{2}H, Alaβ, Ileδ1, Leuδ2, Valγ2-[$^{13}CH_3$]} Gα·GDP. The spectrum was recorded at 20 °C with a Bruker Avance 600 spectrometer. (b) ^{1}H-^{13}C HMQC spectrum of 200 μM {u-^{2}H, Alaβ, Ileδ1, Leuδ2, Valγ2-[$^{13}CH_3$]} Gα·GDP in the presence of 300 μM GoLoco14. The spectrum was recorded at 20 °C with a Bruker Avance 800 spectrometer.

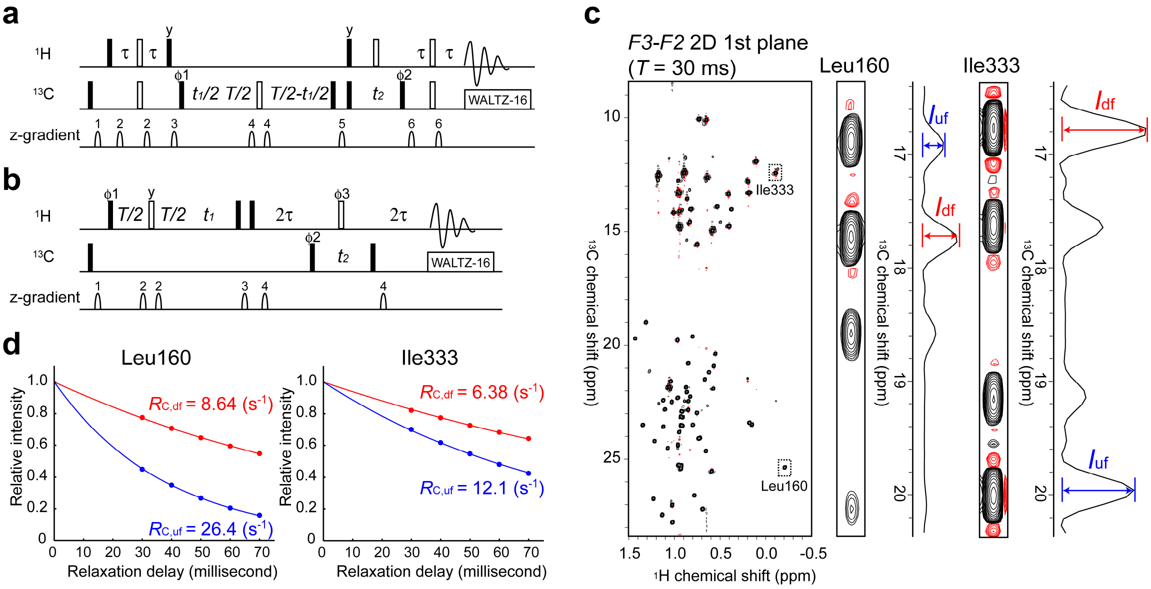

Supplementary Figure 3 Measurements of ^{13}C and ^{1}H CSA values

(a) Pulse sequence for measuring ^{13}C CSA values. Narrow (filled) and wide (open) rectangles represent 90 degree and 180 degree pulses, respectively. Pulse phases are along the x axis, unless otherwise mentioned. The delay τ is set to $1/(4J_{CH})$. ^{13}C decoupling during acquisition is achieved with a 3.3-3.6 kHz WALTZ-16 field [2]. φ1 = (x, -x), φ2 = (x, x, -x, -x), rec = (x, -x, -x, x) Quadrature detection in the F1 dimension is achieved *via* states-TPPI of phase φ1, and quadrature detection in the F2 dimension is achieved *via* states-TPPI of phase φ2[3]. Gradient strengths (G/cm) are: g1 = (2.0 G/cm, 2 ms); g2 = (2.0 G/cm, 0.5 ms); g3 = (7.5 G/cm, 1 ms); g4 = (15 G/cm, 0.5 ms) ; g5 = (-5.0 G/cm, 1 ms) ; g6 = (2.0 G/cm, 0.5 ms). (b) Pulse sequence for measuring ^{1}H CSA values. The delay *τ* is set to $1/(4J_{CH})$. φ1 = (x), φ2 = (x, -x), φ3= (x, x, y, y, -x, -x, -y, -y), rec = (x, -x, -x, x). Quadrature detection in the F1 dimension is achieved *via* states-TPPI of phase φ1, and quadrature detection in the F2 dimension is achieved *via* states-TPPI of phase φ2. Gradient strengths (G/cm) are: g1 = (15

G/cm, 1 ms); g2 = (15 G/cm, 0.5 ms); g3 = (15 G/cm, 0.5 ms); g4 = (20 G/cm, 0.5 ms). (c) The ^{1}H (F3)-^{13}C (F2) first plane of the 3D spectrum measuring the ^{13}C CSA values using the pulse sequence of (a), recorded on the {u-^{2}H, Ileδ1, Leuδ2, Valγ2-[$^{13}CH_3$]} MBP sample (left). The relaxation delay is set to 30 ms. ^{1}H (F3)-^{13}C (F1) planes and 1D cross-sections of Leu160 and Ile333 are shown (right). (d) Plots of the intensities of the up-field signals (I_{uf}, blue) and the down-field signals (I_{df}, red) in Leu160 and Ile333 against the relaxation delay. The relaxation rates were obtained by fitting the intensity ratios to an exponential decay function.

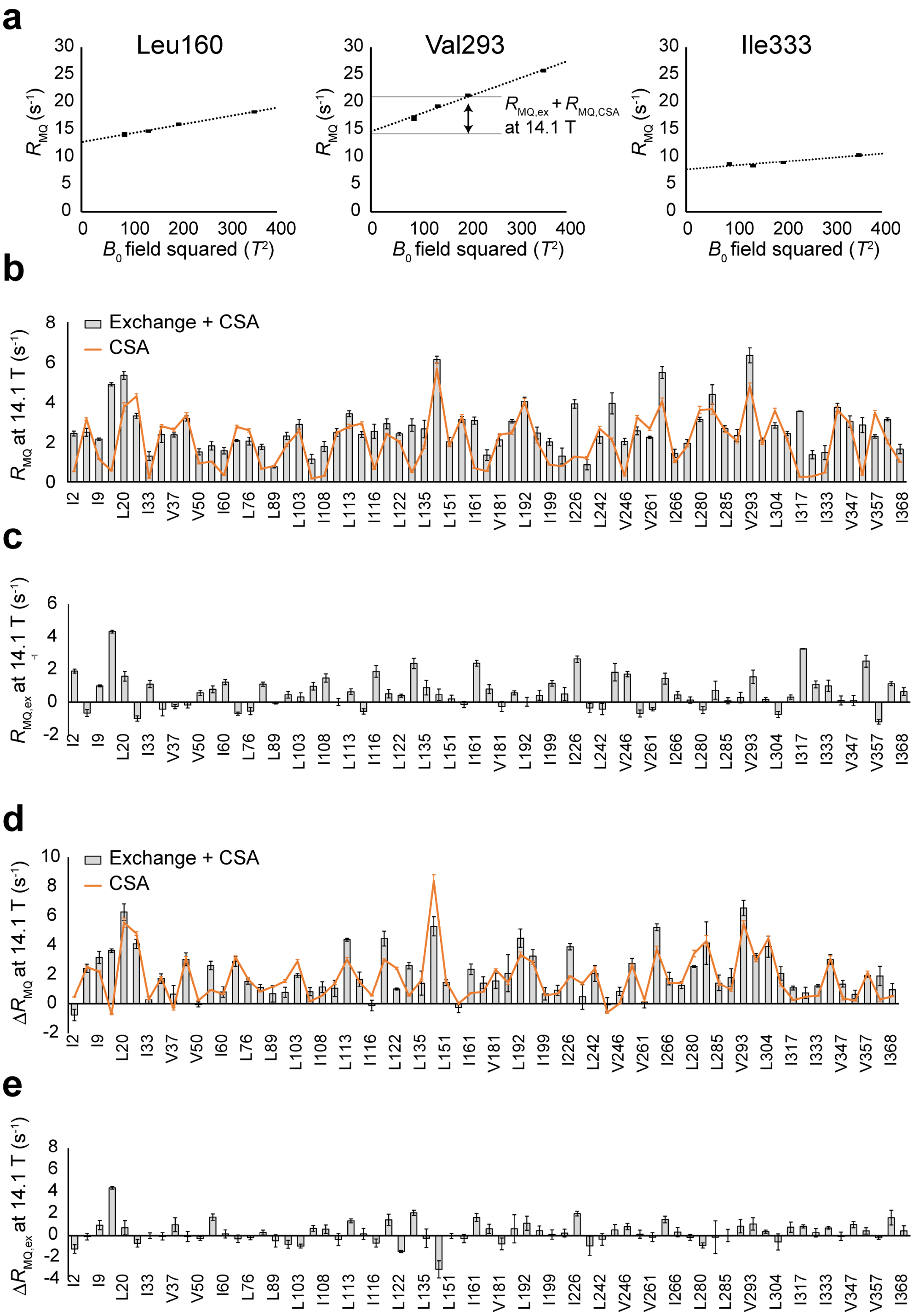

Supplementary Figure 4 Magnetic field-dependent MQ relaxation analyses of MBP

(a) Plots of the R_{MQ} rates of Leu160, Val293, and Ile333 against the square of the static magnetic fields. The sum of the $R_{MQ,CSA}$ and $R_{MQ,ex}$ rates of Val293 at 14.1 Tesla (600 MHz ^{1}H frequency) is shown. (b) Plots of the sum of the $R_{MQ,CSA}$ and $R_{MQ,ex}$ rates (gray bars) and the $R_{MQ,CSA}$ rates (orange line). (c) Plot of the $R_{MQ,ex}$ rates. (d) Plots of the sum of the $\Delta R_{MQ,CSA}$ and $\Delta R_{MQ,ex}$ rates (gray bars) and the $\Delta R_{MQ,CSA}$ rates (orange line). (e) Plot of the $\Delta R_{MQ,ex}$ rates. The results from the Ileδ1, Leuδ2, Valγ2 methyl groups in MBP at 14.1 Tesla (600 MHz ^{1}H frequency) are shown. The error bars represent standard deviation (SD) of fitting errors, estimated from Monte Carlo simulations using uncertainties in peak intensities.

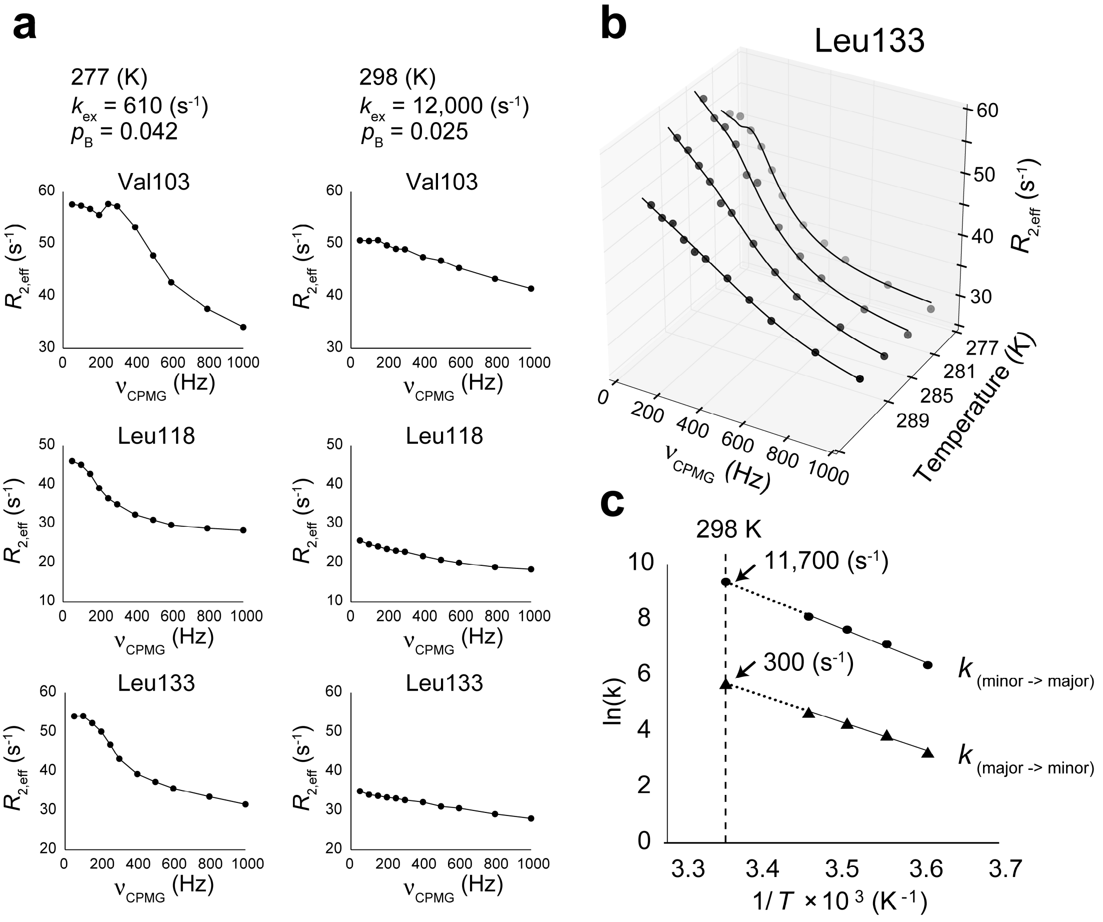

Supplementary Figure 5 CPMG RD experiments of T4L

(a) SQ CPMG RD profiles of Val103, Leu118, and Leu133, measured at 277 K (4 °C) and 298 K (25 °C) (right). ν_{CPMG} was varied from 50 to 1,000 Hz, and the constant relaxation delay was set to 40 ms. The measurements were performed at a static magnetic field strength of 14.1 T (600 MHz ^{1}H frequency). (b) The temperature dependence of the SQ CPMG RD profiles of Leu133. The temperature was varied from 277 K (4 °C) to 289 K (16 °C). The fitted curves are shown as solid lines. (c) Linear Arrhenius plots of the forward and reverse rate constants. The rates at 298 K were obtained *via* extrapolation.

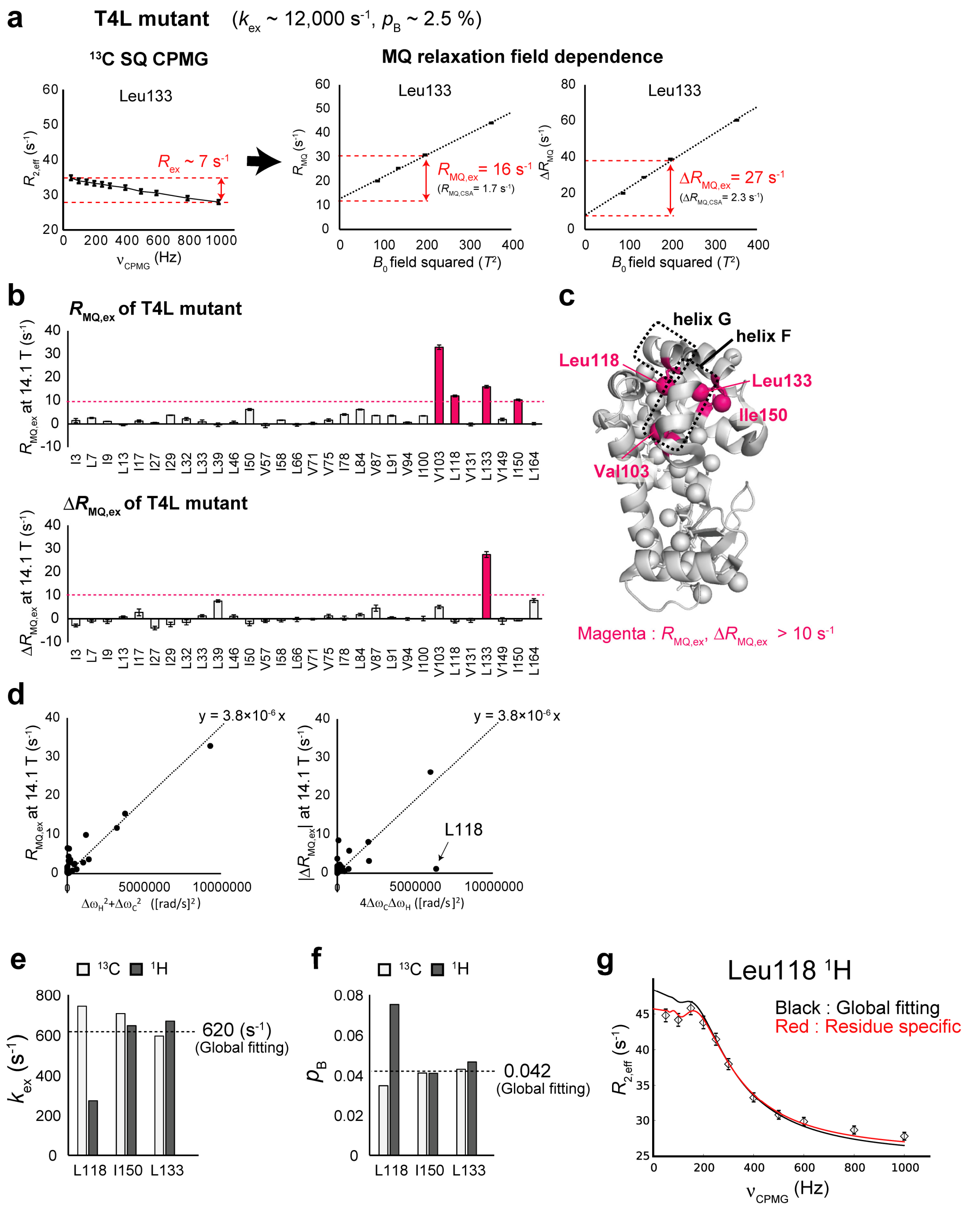

Supplementary Figure 6 Magnetic field-dependent MQ relaxation analyses of T4L

(a) The SQ CPMG RD profile (left) and the plot of the R_{MQ} (middle) and ΔR_{MQ} (right) rates

against the square of the static magnetic field of Leu133. The change in the effective relaxation rates in the SQ CPMG RD profile is shown, and the chemical exchange contributions to the MQ relaxation rates at 14.1 Tesla (600 MHz ^{1}H frequency) are shown. The experiments were performed at 298 K. (b) Plots of the $R_{\mathrm{MQ,ex}}$ (top) and $\Delta R_{\mathrm{MQ,ex}}$ (bottom) rates of the Ileδ1, Leuδ2, and Valγ2 methyl groups of T4L. The methyl groups with significant chemical exchange contributions larger than 10 s^{-1} are colored magenta. The error bars represent SD of fitting errors, estimated from Monte Carlo simulations using uncertainties in peak intensities. (c) Mapping of the methyl groups with significant chemical exchange contributions on the crystal structure of the T4L mutant (PDB ID: 3DMV)[4]. Methyl groups with chemical exchange contributions larger than 10 s^{-1} are colored magenta. (d) Linear correlation plots of the $R_{\mathrm{MQ,ex}}$ (left) and $\Delta R_{\mathrm{MQ,ex}}$ (right) rates obtained from the magnetic field-dependence, against the MQ chemical shift differences, calculated using the results of the CPMG RD experiments. (e, f) Plots of the k_{ex} (e) and p_{B} (f) values obtained by fitting the ^{13}C and ^{1}H dispersion profiles separately. The values obtained from the global fitting procedure are shown. (g) Fitting curves of the ^{1}H dispersion profile of Leu118. The fitting curve obtained from the global fitted k_{ex} and p_{B} values is shown as a black line, and that obtained from the residue-specific k_{ex} and p_{B} values is shown as a red line.

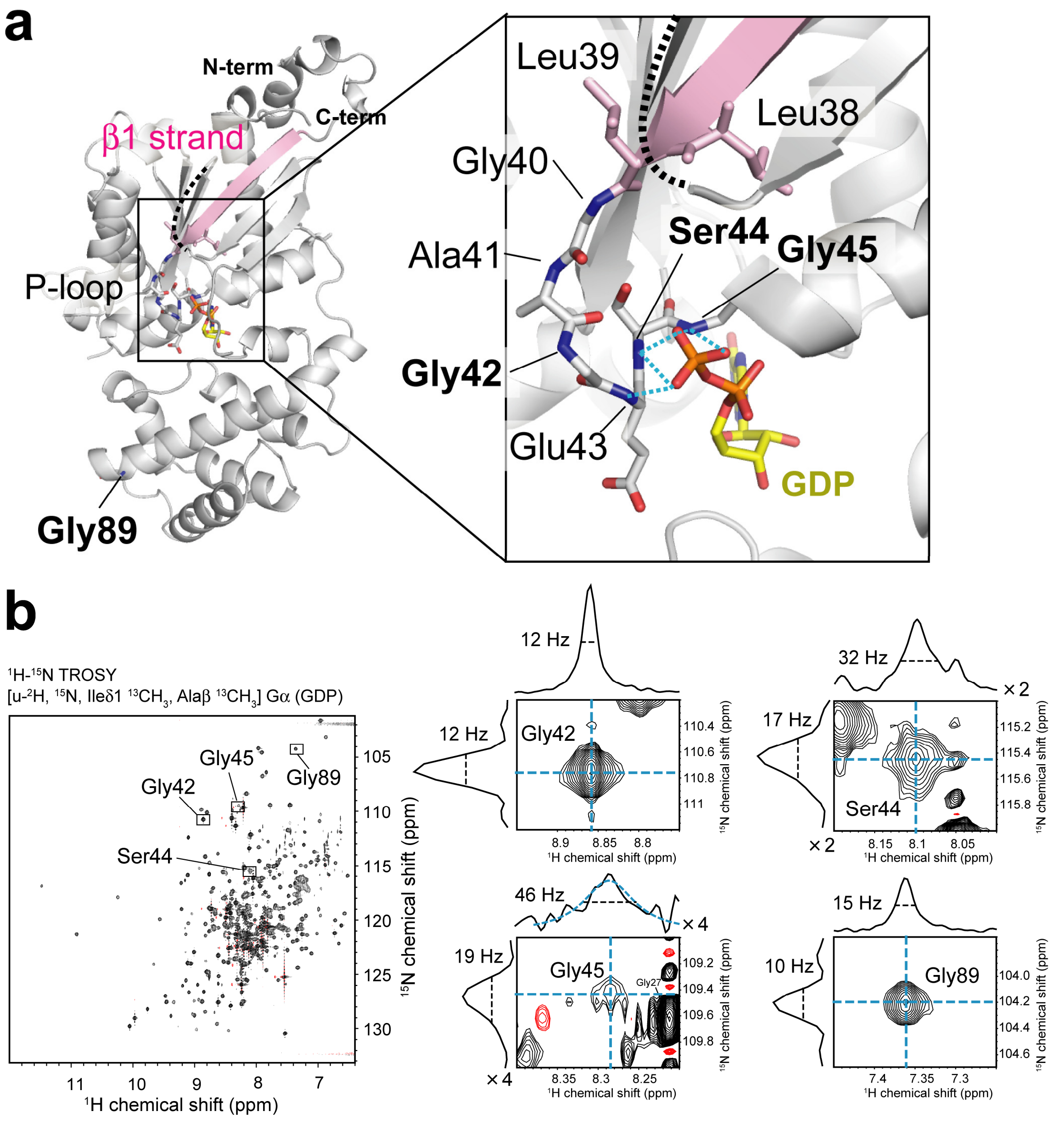

Supplementary Figure 7 Line width analyses of ^{1}H-^{15}N resonances from P-loop residues

(a) Crystal structure of Gα·GDP (PDB ID: 1GDD)[1]. Residues 38-45 (β1 strand and P-loop) and Gly89 (αA helix) are shown as sticks. (b) ^{1}H-^{15}N TROSY spectrum of {u-^{2}H,^{15}N, Ileδ1, Alaβ-[^{13}CH$_3$]} Gα·GDP recorded at 20 °C with a Bruker Avance 600 spectrometer. Line widths of Gly42, Ser44, Gly45, and Gly89 in the ^{1}H and ^{15}N dimensions are shown. The ^{1}H

line widths are significantly broadened in Ser44 and Gly45, with amide groups that form direct interactions with the β-phosphates of the bound GDP, as compared to those in Gly42, with an amide group that does not form direct interactions with the bound GDP, and Gly89, which is located on the helical domain. The data set comprised [220, 2048] complex points in the [^{15}N, ^{1}H] dimensions with the corresponding acquisition times of [106 ms, 57 ms].

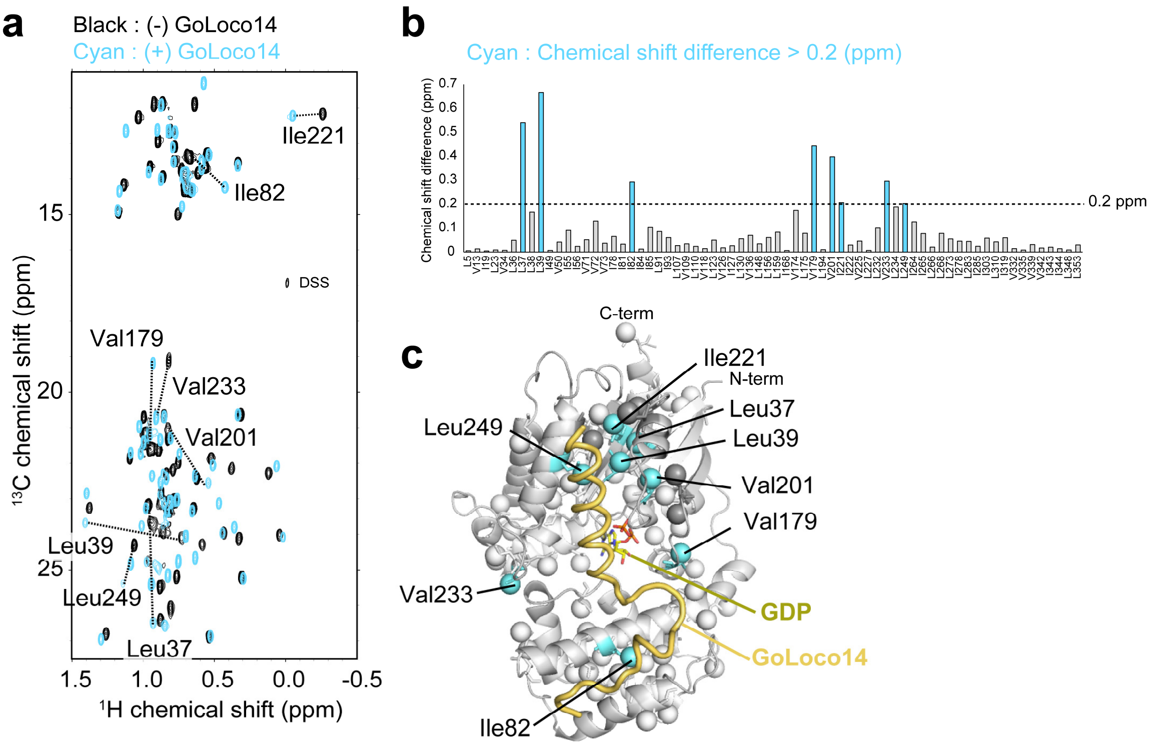

Supplementary Figure 8 Chemical shift differences induced by GoLoco14 binding

(a) Overlay of the ^{1}H-^{13}C HMQC spectra of 200 μM {u-^{2}H, Ileδ1, Leuδ2, Valγ2-[$^{13}CH_3$]} Gα·GDP in the presence (cyan) and absence (black) of 300 μM GoLoco14. The resonances with significant chemical shift differences are labeled. (b) Plot of chemical shift differences induced by GoLoco14 binding. The averaged chemical shift differences are calculated by the equation, $\Delta\delta = [(\Delta\varpi_H^2 + (\Delta\varpi_C/5.8)^2]^{0.5}$. The methyl groups with chemical shift differences larger than 0.2 ppm are colored cyan. (c) The residues with significant chemical shift differences are mapped on the crystal structure of GoLoco14-Gα·GDP (PDB ID: 1KJY)[5]. The methyl groups with chemical shift differences larger than 0.2 ppm are colored cyan. The methyl groups with no data are colored gray.

Supplementary References

1. Mixon, M. B. *et al.* Tertiary and quaternary structural changes in $G_{i\alpha1}$ induced by GTP hydrolysis. *Science* **270,** 954–60 (1995).

2. Shaka, A. J., Keeler, J., Frenkiel, T. & Freeman, R. An improved sequence for broadband decoupling: WALTZ-16. *J. Magn. Reson.* **52,** 335–338 (1983).

3. Marion, D., Ikura, M., Tschudin, R. & Bax, A. Rapid recording of 2D NMR spectra without phase cycling. Application to the study of hydrogen exchange in proteins. *J. Magn. Reson.* **85,** 393–399 (1989).

4. Liu, L., Baase, W. A. & Matthews, B. W. Halogenated Benzenes Bound within a Non-polar Cavity in T4 Lysozyme Provide Examples of I□S and I□Se Halogen-bonding. *J. Mol. Biol.* **385,** 595–605 (2009).

5. Kimple, R. J., Kimple, M. E., Betts, L., Sondek, J. & Siderovski, D. P. Structural determinants for GoLoco-induced inhibition of nucleotide release by Gα subunits. *Nature* **416,** 878–81 (2002).
